# In Memoriam: Katrin Susanne Kohl (1964–2018)

**DOI:** 10.3201/eid2501.180961

**Published:** 2019-01

**Authors:** Nina Marano, Stephen H. Waterman

**Affiliations:** Centers for Disease Control and Prevention, Atlanta, Georgia, USA (N. Marano);; Centers for Disease Control and Prevention, San Juan, Puerto Rico, USA (S.H. Waterman)

**Keywords:** Katrin Susanne Kohl, Centers for Disease Control and Prevention, epidemiology, vaccines, immunization, quarantine, in memoriam

Katrin Susanne Kohl, MD, PhD, MPH, who served over a decade as Deputy Director of the Division of Global Migration and Quarantine, National Center for Emerging and Zoonotic Infections, at the Centers for Disease Control and Prevention (CDC; Atlanta, Georgia, USA), died suddenly and unexpectedly at her home in Atlanta on May 20, 2018, at the age of 54. She was a beloved physician epidemiologist leader at CDC who made major contributions in international health and vaccine safety.

While at CDC’s Immunization Safety Branch starting in 2000, Kohl brilliantly succeeded in launching and coordinating The Brighton Collaboration, an international effort of >800 participants in 80 countries to enhance vaccine safety. The collaboration was a new global initiative to improve the rigor of immunization safety science at a time of increasing public controversy and vaccine hesitancy. She worked tirelessly to promote the new collaboration among key policymakers in the global immunization community, resulting in publication of the first set of Brighton case definitions in the journal Vaccine.

Kohl was born in Duisburg, Germany, in 1964. Her Austrian father was a prominent architect there. She attended medical school in Graz, Austria, and completed her MD in 1991 and PhD in 1993 at the Free University of Berlin, Germany. In 1996, she earned her MPH from Tulane University School of Public Health and Tropical Medicine (New Orleans. LA, USA). She joined CDC in 1997 as an Epidemic Intelligence Service Officer assigned to the Louisiana Department of Health, where her work focused mainly on infectious disease epidemiology and sexually transmitted diseases. Tom Farley, her Epidemic Intelligence Service supervisor and later the New York City Commissioner of Health, remarked that the Louisiana staff fell in love with this German woman who made disease investigation a happy and exhilarating experience.

At the Division of Global Migration and Quarantine, working with the division’s director Martin Cetron, she participated in CDC quarantine and border health responses for Middle East respiratory syndrome, the 2009 (H1N1) influenza pandemic, the 2014–2015 Ebola epidemic in West Africa, and the 2016 Zika epidemic. She also played a key role in strengthening a new unit addressing United States–Mexico binational and border health. Kohl was the division’s champion for helping to implement the World Health Organization’s 2005 International Health Regulations, skillfully educating staff from US federal and state agencies, and liaising with World Health Organization member states and regional offices to help implement enhanced transparency and collaboration in international disease and outbreak reporting. Kohl also published in and reviewed articles for Emerging Infectious Diseases.

In addition to her scientific accomplishments, Kohl will be remembered for her passionate, caring, and ebullient personality, which made her such an effective ambassador, bringing together persons with diverse scientific viewpoints to reach technical and public health policy consensus. She will be greatly missed by her husband, Gene Spiegel; her son, Alexander, and daughter, Clara; her mother, Christel Kohl; her brother, Christian; and her many colleagues at CDC and around the world.

**Figure:**
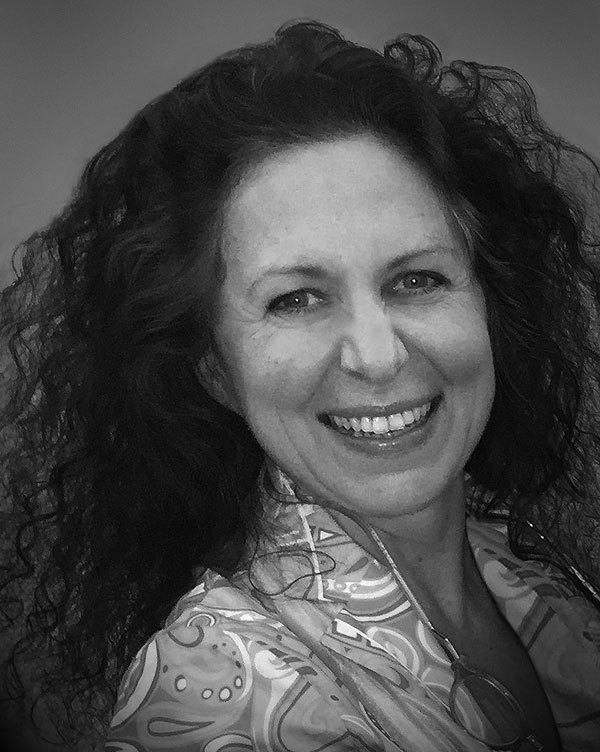
Katrin Susanne Kohl.

